# Effect of swelling agent treatment on grape fruit quality and the application of electronic nose identification detection

**DOI:** 10.3389/fpls.2023.1292335

**Published:** 2024-01-17

**Authors:** Jianlei Qiao, Guoqiang Su, Liang Yuan, Lin Wu, Xiaohui Weng, Shuang Liu, Yucai Feng, Dan Jiang, Yuxuan Chen, Yuan Ma

**Affiliations:** ^1^ College of Horticulture, Jilin Agricultural University, Changchun, China; ^2^ School of Mechanical and Aerospace Engineering, Jilin University, Changchun, China; ^3^ Weihai Institute for Bionics, Jilin University, Weihai, China

**Keywords:** grape, swelling agent, fruit quality, nondestructive testing, electronic nose

## Abstract

The swelling agent is a plant growth regulator that alters the composition and content of nutrients and volatile gases in the fruit. To identify whether grape fruit had been treated with swelling agent, the odor information and quality indexes of grape berries treated with different concentrations of swelling agent were examined by using electronic nose technology and traditional methods. The contents of soluble sugars, soluble solids, soluble proteins and vitamin C were significantly increased in N-(2-chloro-4-pyridyl)-N’-phenylurea (CPPU) treated fruit. The contents of hexanal, (E)-2-hexenal, and nonanal aldehydes decreased significantly. Similarly, the levels of phenyl ethanol, 1-octanol, ethanol, and ethyl acetate alcohols and esters also decreased noticeably. Additionally, the levels of damascenone, linalool, and geraniol ketones and terpenoids decreased. However, the contents of benzaldehyde, D-limonene, acetic acid and hexanoic acid increased. In addition, the electrical signals generated by the electronic nose (e-nose) were analyzed by linear discriminant analysis (LDA), support vector machine (SVM) and random forest (RF). The average recognition rate of SVM was 94.4%. The results showed that electronic nose technology can be used to detect whether grapes have been treated with swelling agent, and it is an economical and efficient detection method.

## 1 Introduction

Grape (*Vitis vinifera L.*) is one of the longest cultivated and most productive fruit tree species in the world and one of the most popular fruit crops. As one of the world’s leading grape producers, China’s viticultural area and grape production have been on the rise. Grapes are popular in the market because of their high nutritional value (Samoticha et al., 2017; Silva et al., 2018; Leng et al., 2023), such as vitamins and mineral. For table grapes, grape fruit aroma is a secondary metabolite, which has an important impact on grape quality (Yao et al., 2021) and is an important characteristic that determines consumer preference (Ji et al., 2019). One of the most important factors affecting the aroma profile of a particular grape variety is the stage of ripening (Yao et al., 2021). Studies have shown that grape fruit contains volatile aromatic substances, such as esters, alcohols, aldehydes and others (Diéguez et al., 2003). Consequently, the aromas of table grapes are very important to study.

Plant growth regulators, classically referred to as phytohormones, are a group of chemically diverse compounds that govern or influence both plant developmental programs and responses to inner and outer cues at minute concentrations (Castro-Camba et al., 2022). N-(2-chloro-4-pyridyl)-N’-phenylurea (CPPU) is a plant growth regulator commonly used in horticultural crop production to regulate the growth and development of certain organs of crops. Huitrón et al (Huitrón et al., 2007). indicated that CPPU-treated watermelons had a lower accumulation of sugars than those treated with 2,4-D. A decrease in total soluble solids was also reported in CPPU-treated muskmelon (Hayata et al., 2000) and watermelon (Lopez-Galarza et al., 2004). Rational use of this swelling agent could promote the division, differentiation and enlargement of fruit cells, as well as to improve crop yield (Jing et al., 2013; Matsuo et al., 2013). However, irrational use will cause fruit appearance deformity, flavor deterioration, accelerated fruit softening, tree potential decline and other negative effects (Antognozzi et al., 1996; Qing-Ping, 2001), especially on fruit quality, including the reduction of anthocyanin, vitamin C and sucrose content, as well as the increase of bitterness (Hayata et al., 2000; Qian et al., 2018; Luo et al., 2020). Studies have shown that swelling agent treatment can reduce internal physiological indicators such as soluble sugar, acidity and firmness of grapes (Wang et al., 2017). There is often an unreasonable phenomenon of abuse of swelling agent in agricultural production. Some farmers excessively pursue large granulation of fruit and overspray these kinds of compounds, which seriously affects the taste and nutrition of grapes, resulting in grape distortion, a large number of bad and cracked fruit, and seriously affects the quality of grapes. The abuse of swelling agent leads to uneven product quality and market price confusion, which has become an important factor restricting the healthy development of the grape industry. Therefore, it is of great significance to explore some rapid and efficient methods to identify whether grape fruit has been treated with swelling agent.

The current methods for detecting the quality of grape fruits mainly include instrumental analysis, chemical analysis and sensory evaluation. With the increase of CPPU concentration, the size, color, shape, nutrient composition and content of grapes will change to some extent (Wang et al., 2017), such as the size and shape of grapes increase and the content of nutrients change, which provides a theoretical basis for the application of physicochemical index detection method in the detection of grape fruits treated with swelling agent. However, these methods are time-consuming and laborious, which make it difficult to meet the practical testing requirements. With the advantages of fast, sensitive, real-time, and nondestructive testing due to simple sample preparation, electronic noses offer a rapid and nondestructive alternative to traditional methods that rely on lengthy laboratory processing (Peris & Escuder-Gilabert, 2016). At present, electronic nose detection technology has been widely used in vegetable, fruit and other crops maturity, freshness, damage identification and other fields (Qiu et al., 2017; Xing et al., 2018; Chen et al., 2019), and the e-nose technique combined with different pattern recognition methods (e.g., LDA.RF and SVM) can rapidly identify samples (Qiao et al., 2022). Gas chromatography and mass spectrometry are mainly used to study the volatile gases of grape (Ji et al., 2019; Yao et al., 2021), and these research methods have disadvantages such as time-consuming and laborious, the next step will be to explore the electronic nose technique combined with LDA, RF and SVM methods for rapid detection of grape fruit quality.

Previous studies on the combination of electronic nose technology for the detection of swelling agent-treated grape fruits are relatively scarce, and the composition and content of volatile gases in treated fruits may be different compared with naturally ripened (un-expanded) fruits. Based on this, the present study is intended to detect and analyze the aroma components of grape berries treated with different concentrations of swelling agent using electronic nose technology and traditional physicochemical experimental methods, in order to quickly identify whether grape berries have been treated with swelling agent or not, and to provide a new method for the quality testing of grape berries in the market.

## 2 Materials and methods

### 2.1 Experimental design

The grape cultivation experiment was carried out in the orchard of Jilin Agricultural University (43°48′ N, 125°25′ E), Changchun, China. The materials used in the experiment were “Xiangti” grape plants, with the same number of years of growth as well as planting culture, free of pests and diseases. Field soil fertilization management and pest control were carried out under controlled conditions in the greenhouse and in a conventional manner. This base is a solar greenhouse, planted to avoid rain. The spacing between plants and rows was set to 2.0m×2.0m. The swelling agent used was N-(2-chloro-4-pyridyl)-N ‘-phenylurea (CPPU). The first spray treatment was carried out on June 7, 2021 (when the grapes grew to the size of soybean grains, and the second spray treatment was carried out on June 14. The swelling agent with different concentration gradients was sprayed between each treatment, and water was sprayed as the control (CK). The amount of spraying was determined by the fact that there was no liquid dripping on the peel surface. The CK and treatment group were treated with the same water and fertilizer management. CPPU treatment concentrations were 2 mg/L, 4 mg/L and 6 mg/L which were recorded sequentially as treatment A, treatment B, and treatment C. Each treatment was repeated 3 times. At harvest, samples of grapes free of pests or damage were selected from the same orchard, with 40 samples per treatment. Following the selection of the fruits for each treatment, an electronic nose was used to identify the odor information of the fruit before the fruit quality indexes were determined. All samples were evaluated at room temperature to lower experimental error.

### 2.2 E-Nose detection

**Figure 1:**
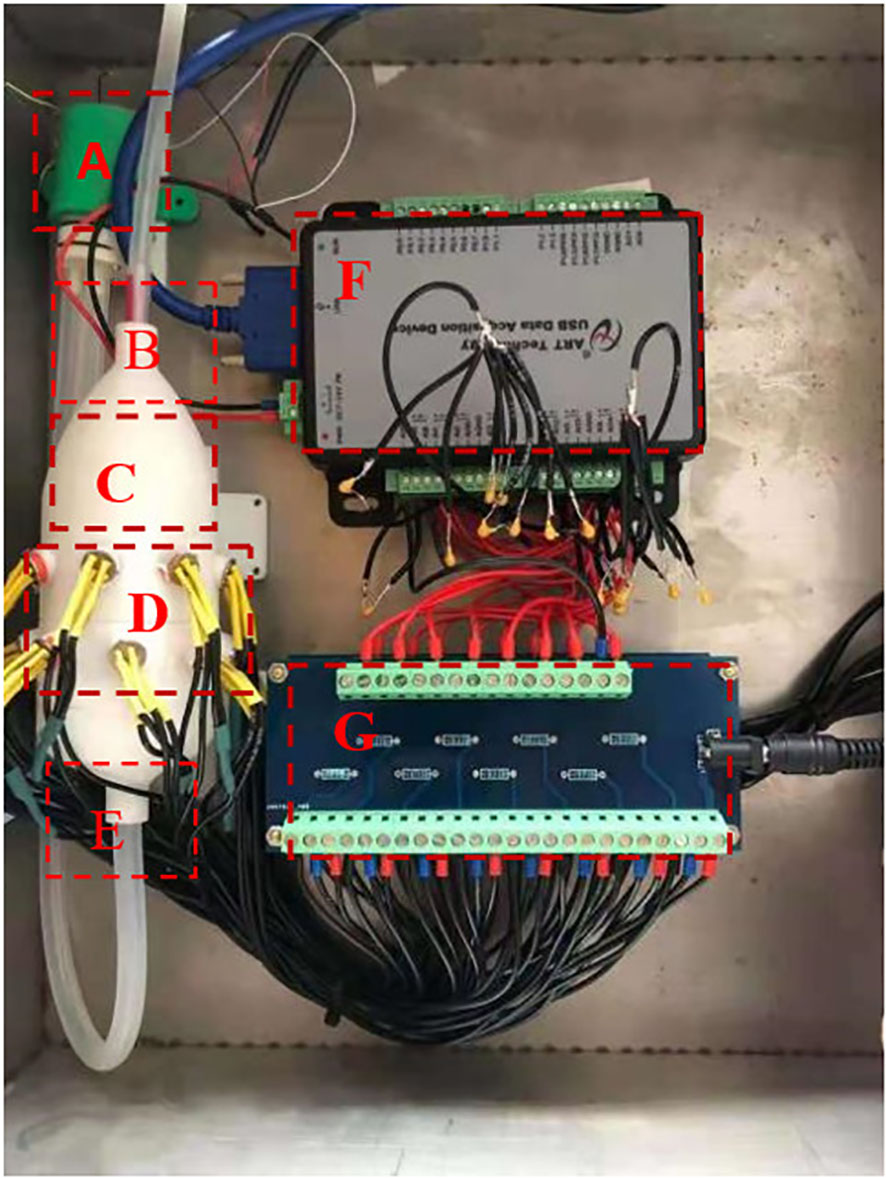
Electronic nose system diagram. **(A)** the miniature air pump; **(B)** the air inlet; **(C)** the sampling chamber; **(D)** the air-sensitive sensor array; **(E)** the air outlet; **(F)** the data acquisition card; **(G)** the test circuit for the electronic nose.

An electronic nose system created (Jilin University, Changchun, China) in the lab was used to examine the materials. A data gathering card, a test circuit, a tiny air pump, a sampling chamber, and a number of gas-sensitive sensors make up the system. The physical diagram is shown in **Figure 1**. The gas sensing system array is composed of 16 different metal oxide semiconductor (MOS) sensors, and each sensor is sensitive to a specific class of volatile chemicals in the sample gas. The 16 MOS sensors are listed in **Table 1** with a brief description of the primary application for each sensor and the manufacturer of each sensor.

**Table 1:** Application of sensors used in the sensor array.

number	Sensors	Main applications	Manufacturer and country
S1	TGS2612	Hydrocarbons, Methane, Liquefied Petroleum Gas	Figaro, Osaka, Japan
S2	GSBT11	Volatile Organic Compounds (VOC), some sulfur- and nitrogen-containing inorganic gases	Ogam, Jeollanam-do, Korea
S3	WSP2110	Benzene, Toluene, Formaldehyde	Winsen, Zhengzhou, China
S4	MP135	Alcohols, ketones, benzene ring-containing compounds, nitrogen-containing compounds	Winsen, Zhengzhou, China
S5	MS1100	VOCs, Toluene, Benzene, Formaldehyde,	Ogam, Jeollanam-do, Korea
S6	MP901	Alcohol, Cigarette Smoke, Formaldehyde, Toluene, Benzene, Acetone	Winsen, Zhengzhou,China
S7	TGS2611	Alcohols, hydrocarbons, inorganic gases	Figaro, Osaka, Japan
S8	TGS2620	VOCs, Alcohols, Organic Solvents Steam	Figaro, Osaka, Japan
S9	TGS2602	Ammonia, Hydrogen Sulfide (high sensitivity to VOC and odorous gases)	Figaro, Osaka, Japan
S10	TGS2610	Alcohols,Butane, Liquid Petroleum Gas, Propane	Figaro, Osaka, Japan
S11	TGS2600	Ethanol, Hydrogen, Hydrocarbons, etc.	Figaro, Osaka, Japan
S12	TGS2603	Trimethyl Amine, Methyl Mercaptan, Hydrogen Sulfide, etc.	Figaro, Osaka, Japan
S13	MP-4	Methane, alcohols, hydrocarbons	Winsen, Zhengzhou, China
S14	MP-2	Methane, Alcohols, Hydrocarbons	Winsen, Zhengzhou, China
S15	MP-5	Most of the VOCs, Propane	Winsen, Zhengzhou, China
S16	MP-7	Most VOCs, Nitric Oxide	Winsen, Zhengzhou, China

**Figure 2:**
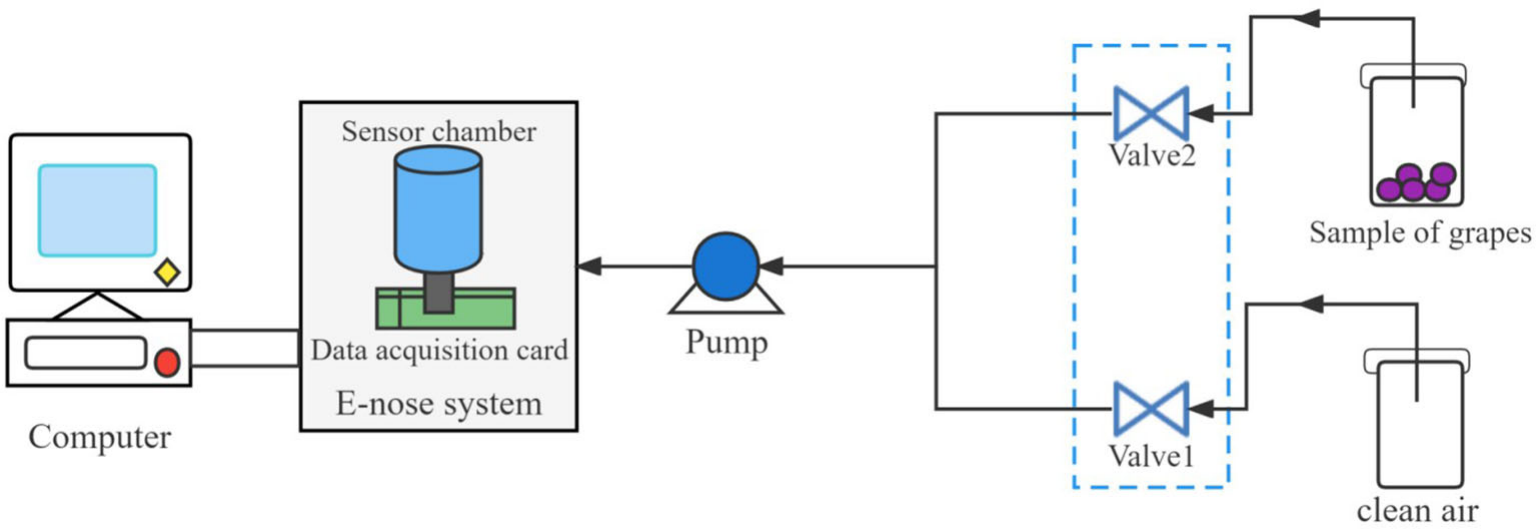
Electronic-nose device schematic diagram.

For the purpose of the experiment, the fruits from each treatment were randomly divided into 40 groups each containing 5 fruits, and the experiment yielded a total of 160 sets of electronic nose sampling data from grape samples at different treatment stages (40 replicates × 4 treatments). **Figure 2** illustrates the schematic diagram of the electronic nose measurement process in the experiment. The electronic nose was activated 120 minutes before detection to ensure that the sensor surface was heated to operating temperature and the gas path was cleaned with pure air. During the cleaning process, the gas path and sensor chamber were filled with clean air to control the normalization of the sensor signal. During the detection process, each group of grapes was placed in a 300 ml glass beaker and sealed with plastic wrap for 15 minutes, in order to ensure that the volatile chemicals in the grapes filled the beaker and equilibrated. Sample gas was then pumped in by headspace aspiration and flowed through the sensor array at a rate of 300 mL/min. As the sample gas was introduced into the sensor array, the test circuit converted the resulting conductance change to a voltage change (V), which was recorded as the response of the electronic nose sensor. The sample collection time for this experiment was 50 s, and the electronic nose collection frequency was set to 100 Hz. 5000 data were obtained from each sensor during each sample collection. This data is automatically recorded and used for subsequent analysis. After each measurement, the electronic nose sensors were reset and recalibrated with clean gas for 300 s before the next round of headspace sampling.

### 2.3 Determination of fruit quality indexes

After non-destructive detection by the electronic nose, the fruits were subjected to relevant quality indicators. The soluble sugar content was determined by the anthrone colorimetric method. Measurements were made with specific reference to the method of Magné et al. (Magné et al., 2006). Titratable acid content was determined concerning GB/T 12456-2008 “Acid-base titration method in the determination of total acid in food (Zahedipour et al., 2019; Gao and Xu, 2022)”. Sugar-acid ratio: The sugar-acid ratio is expressed as the ratio of soluble sugar content to titratable acidity content. The content of soluble protein was determined concerning GB 5009.5-2016 “Determination of Protein in Food Safety National Standard”. The vitamin C content was determined regarding GB 5009.86-2016 “Determination of Ascorbic Acid in Food” of the National Food Safety Standard. Determination of soluble solids content: measured by hand-held refractometer. Samples from each treatment were randomly selected for the determination of quality indicators, and each indicator was repeated three times, and the test results were averaged. Soluble solids content was determined with reference to the method of Zhu et al (Zhu et al., 2020). To process and analyze the experimental data, we utilized Microsoft Excel 2019 (USA) and SPSS26.0 statistical software (IBM, USA). The Duncan’s new complex polar difference method was used to test the significance of the differences between treatments (P<0.05). The purity of the chemical substances used for the determination of fruit quality indexes in this study was analytical pure and was provided by Changchun Anmei Biotechnology Co (Changchun, China).

### 2.4 Determination of volatile compounds in fruits

The content of aroma substances was determined by solid-phase headspace microextraction (HS-SPME) combined with gas chromatography-mass spectrometry (GC-MS) (Pu et al., 2022; Ju et al., 2023).

Extraction of volatile substances: The 15 g of grape fruit tissue was ground into powder with the treatment of liquid nitrogen and transferred to a centrifuge tube containing 1 g of cross-linked polyvinyl pyrrolidone and 0.5 g of D-gluconolactone. The centrifuge tubes were placed in the refrigerator at 4°C for 120 min. and then centrifuged at 4°C for 15 min at 1000 r·min^-1^ to obtain clarified grape juice. It was took 5 mL of grape juice into a 15 mL headspace vial, added 1 g of sodium chloride, 5 μL of internal standard 2-octanol (0.45mg/mL) and magnetic rotor, and quickly tighten the cap, insert the SPME fiber (DVB/CAR/PDMS 50/30 µm) into the sample headspaced vial and placed it in a magnetic stirrer at 60°C for 30 min. After the adsorption process, remove the SPME fiber and then insert it into the inlet of the gas chromatograph. It was then resolved at a temperature of 200°C for a duration of 5 minutes.

Gas chromatography separation conditions: Chromatographic columns: HP-INNO-Wax capillary column (length 30 m, inner diameter 0.25 mm, liquid film thickness 0.25 μm), carrier gas He (99.99%), flow rate 1.10 mL; the sample inlet temperature 200°C, ramp-up procedure: 35°C for 3 min, ramp up to 120°C at a rate of 4°C·min^-1^, hold for 2 min, Increase the temperature at a rate of 10°C-min-1 to 230°C. Mass spectrometry detection conditions: Mass spectrometry detection conditions: GC-MS transmission line temperature of 250°C, EI ion source temperature of 170°C, electron energy of 70eV, photomultiplier voltage of 350V, the mass scan range of 30-350amu.

Qualitative analysis and quantitative analysis: Both NIST08 and RTLPEST3 spectral library searches and information were used for qualitative analysis (Yao et al., 2021). The relative quantification was performed by the internal standard method, using 2-octanol as the internal standard to determine the relative content, calculated as: μg·L^-1^= μg ×L.

### 2.5 Analysis of e-nose data

#### 2.5.1 Feature extraction methods

In the electronic nose data processing process, in order to effectively reduce the time of processing data, in general, the electronic nose data will be signal amplification, filtering, baseline processing and drift compensation and other pre-processing mainly to eliminate or reduce the noise and signal drift caused by various factors in the test process, to achieve the purpose of the electronic nose signal stability. Moreover, in the electronic nose system, multiple gas-sensitive sensors constitute a sensor array, and there is cross-sensitivity between the sensors, so it is usually necessary to standardize the data and eliminate the magnitude of the response data when performing data analysis.

Feature extraction refers to the extraction of a feature matrix from the sensor data according to a certain rule. Previous literature shows that different feature extraction methods have different classification performance (Qiao et al., 2022). Based on the analysis of the electronic nose response signal, the maximum value, average value, integral value and wavelet transform are selected for feature extraction. In this study, all feature values of the data were performed with MATLAB 2013 software.

Maximum value represents the final steady-state characteristic of the entire dynamic response process at final equilibrium, reflecting the maximum variation of the sensor response to odor. It is used as the most common feature extraction method.

Average value is a calculation used to measure the mean value of data. It not only reflects the central tendency of a set of data, but also allows comparing different data and seeing the differences between different data sets.

Integrated value represents the area of the response curve versus the time axis in the response interval, which reflects the overall response of the sensor to the volatile component of the sample to be measured.

Wavelet transform decomposes the original response into low and high frequencies. It has good immunity to interference and is capable of multi-resolution analysis. It also has the ability to characterize local features of the signal in both the time and frequency domains.

#### 2.5.2 Pattern recognition

Pattern recognition means classifying and identifying different types of gas samples and processing and analyzing the information that characterizes things mathematically through computers. In this study, a 10-fold cross-validation method combined with linear discriminant analysis (LDA), support vector machine (SVM), and random forest (RF) methods will be used for pattern recognition accuracy of the sample data (Qiu et al., 2015; Zhu et al., 2020). LDA is a common classification method where the classification results of each group are linearly correlated. LDA is computed using category information and is designed to minimize intra-class ratios and maximize inter-class ratios. The SVM algorithm is a pattern recognition algorithm. SVM was originally developed for linear categorization of separable data, but is also applicable to nonlinear data using kernel functions. The main goal of SVM is to define decision boundaries for different classes of data points using hyperplanes, with edges separating the classes in the hyperplane from the distances from the data set to the nearest point in the data set. RF is a nonparametric, nonlinear classification and regression algorithm. The algorithm is a collection of multiple decision trees, where each tree is classified based on a randomly selected subset of attributes. Majority voting is then used to obtain the final classification result, where the tree with the highest number of classifications is selected. Decision trees have received increasing attention due to the speed with which they can be produced. The analysis of different feature values of the e-nose data by LDA, SVM and RF will investigate how to use the e-nose in combination with the most appropriate recognition algorithm to identify whether the grapes have been treated with swelling agent and to determine the most appropriate feature values for the sample data. In this study, the pattern recognition algorithms (LDA, SVM, and RF) were performed using R language 4.0.2 software (University of Auckland, New Zealand).

## 3 Results

### 3.1 Effects of swelling agent treatment on grape fruit quality indexes

#### 3.1.1 Soluble sugar content, titratable acid content and sugar–acid ratio of fruit

**Figure 3:**
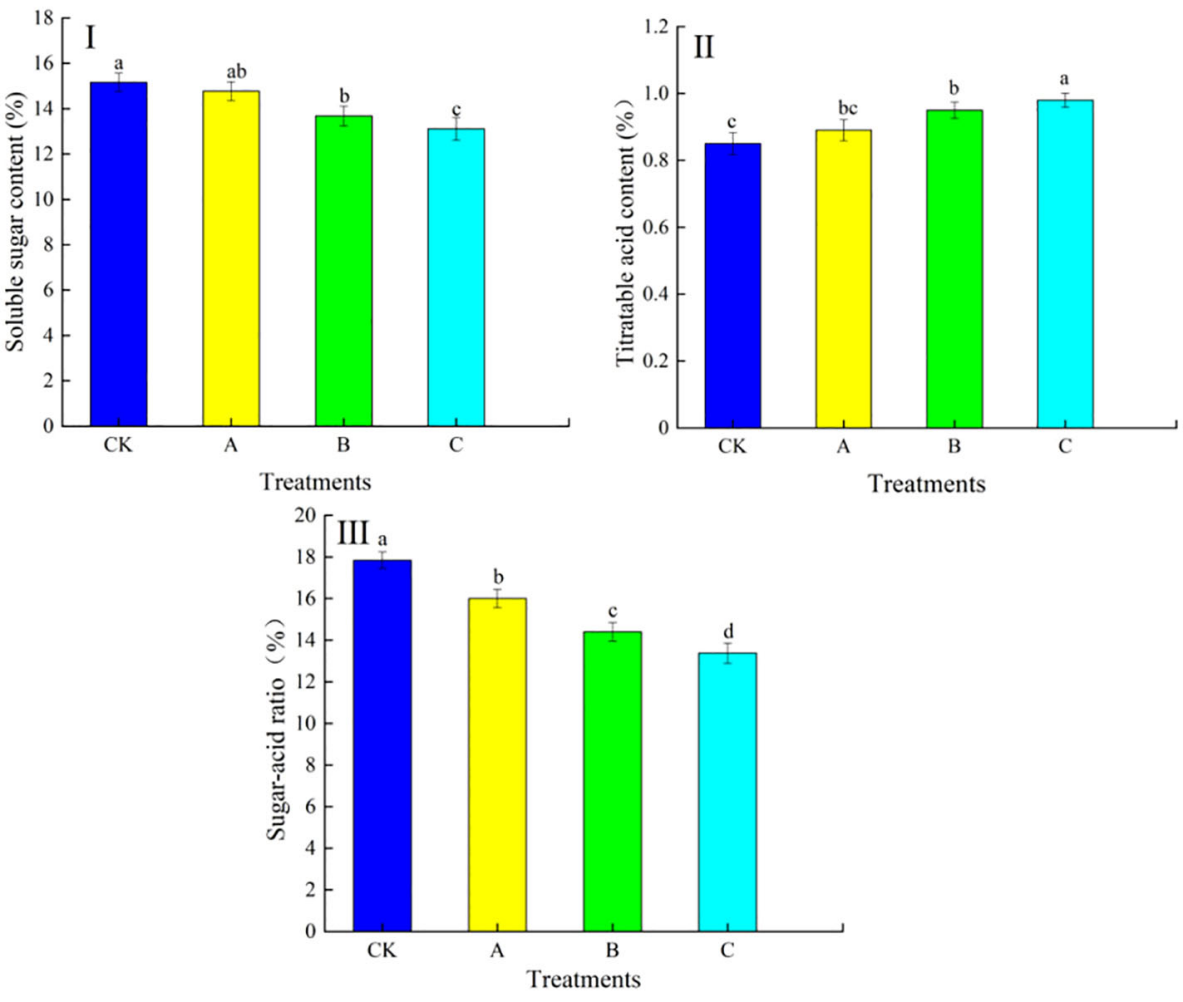
Effect of swelling agent treatment on soluble sugar content, titratable acid content and sugar-acid ratio in fruits. (I) Soluble sugar content, (II) titratable acid content, (III) sugar-acid ratio. Different lowercase letters in the graphs indicate significant differences between treatments (P<0.05).

The differences in soluble sugar contents, titratable acid contents and sugar–acid ratio in grapes under different treatments are shown in **Figure 3**. **Figure 3** showed that the expander treatment had a great influence on the soluble sugar content and titratable acid content in grapes. As shown in **Figure 3 (I)**, the soluble sugar content of treatment A, treatment B and treatment C were lower than the CK. However them, the difference between the soluble sugar content of treatment A and CK did not reach a significant level (P>0.05). Treatment B and treatment C significantly decreased by 9.76% and 13.52%, respectively, compared to CK, and both were significantly lower than CK (P<0.05). As shown in **Figure 3 (II)**, the titratable acid content of treatment A, treatment B and treatment C were all higher than the CK, where the titratable acid content of treatment A was not significantly different compared to CK (P>0.05). The titratable acid content of both treatment B and treatment C was significantly higher than that of CK, 11.76% and 15.29%, respectively, and both were significantly different from CK (P<0.05). As a result, the fruit sugar-acid ratio changed significantly with the swelling agent treatment (**Figure 3III**). The control fruit had the highest soluble sugar content and sugar-acid ratio and better taste compared to treatment A, treatment B and treatment C.

#### 3.1.2 Content of soluble protein, vitamin c, and soluble solids of fruits

**Figure 4:**
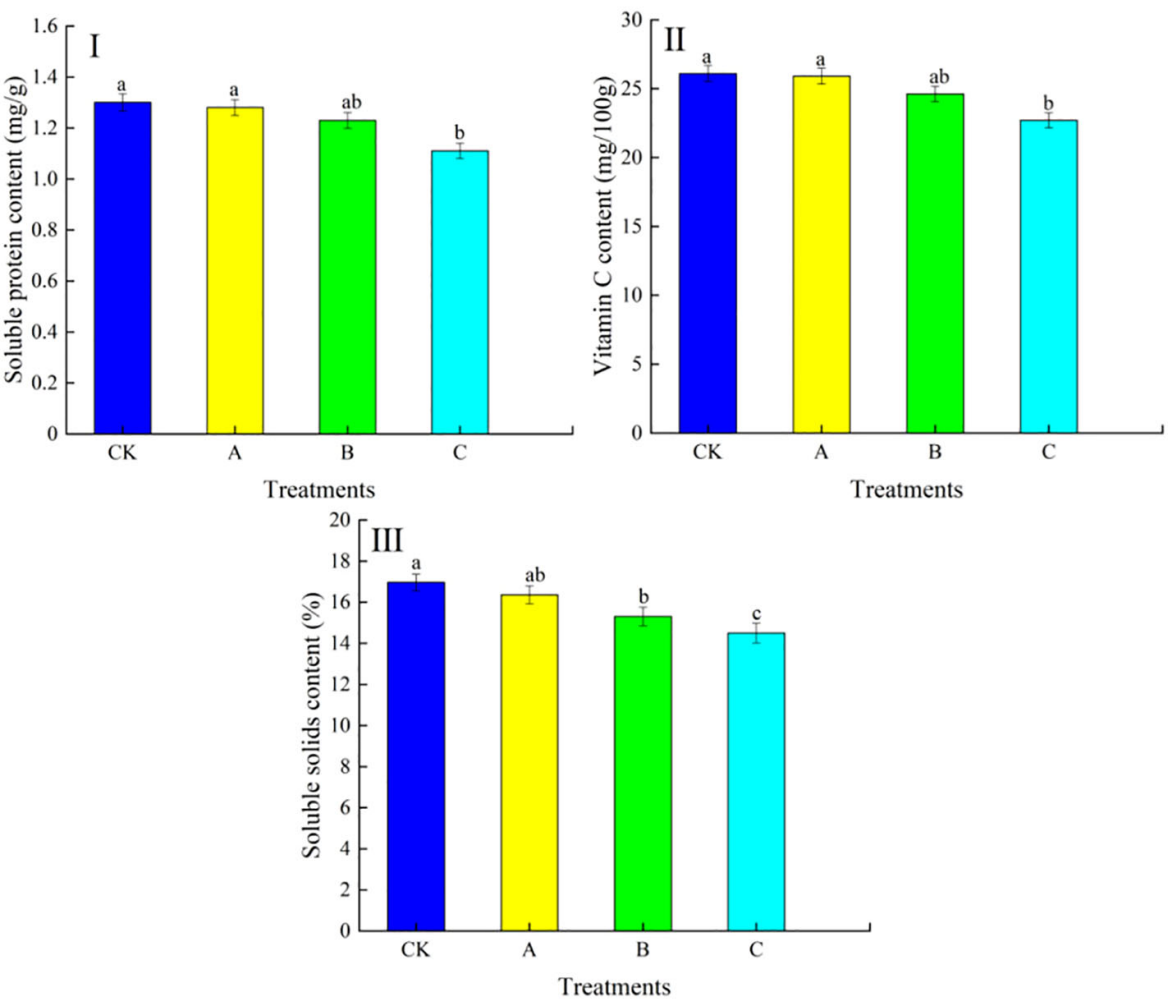
Effect of swelling agent treatment on soluble protein content, vitamin C content and soluble solids content in fruits. (I) Soluble protein content, (II) vitamin C content, (III) soluble solids content. In the graphs, different lowercase letters indicate significant differences between treatments (P<0.05).

The differences in soluble protein content, vitamin C content and soluble solids content in grapes under different treatments are shown in **Figure 4**. As can be seen from **Figure 4 (I)**, it is observed that treatment with swelling agent had a greater effect on soluble protein content in grapes. As the concentration of swelling agent increased, the soluble protein content of the fruits showed a decreasing trend. Among them, no significant change in soluble protein content was observed in treatment A compared with CK. The soluble protein content of treatment B decreased by 5.38% compared with CK, and the difference between treatment B and CK did not reach a significant level (P>0.05). Significantly lower soluble protein content was observed in treatment C compared to CK, with a significant decrease of 14.62% compared to CK. It can be seen from **Figure 4 (II)** that with the increase of the swelling agent concentration, the vitamin C content in the fruit shows a decreasing trend. The changes in treatment A were not significant compared with CK. And the vitamin C content of treatment B decreased by 5.67% compared with CK, and the difference between it and CK did not reach a significant level (P<0.05). Treatment C had significantly lower vitamin C content than CK, with a significant decrease of 13.03% compared to CK. From **Figure 4(III)**, it can be seen that with the increase of swelling agent concentration, the soluble solids content of swelling agent treated fruits were all reduced compared to the control, and the difference between treatment B and treatment C and CK was significant (P<0.05).

### 3.2 Effects of swelling agent treatment on the main volatile substances content in grape fruits

The contents of the main volatile substances in the grapes under different treatment conditions are shown in **Table 2**. The changes in the content of aldehydes showed that the contents of hexanal, (E)-2-hexenal and nonanal in grape fruit were significantly reduced under the swelling agent treatment; the swelling agent treatment caused a significant increase in the content of benzaldehyde in grape fruit, with treatment A, treatment B and treatment C increasing by 19.06%, 23.75% and 31.12%, respectively, compared with the CK; the content of decanal in fruit did not differ significantly between the treatments (P>0.05); the content of (E)-2-heptenal showed an increasing and then decreasing trend with the increase of the swelling agent concentration.

**Table 2:** Effects of swelling agent treatment on the main volatile substances content in grape fruits.

Category	Volatile substance	Content μg·kg^-1^			
		CK	Treatment A	Treatment B	Treatment C
Aldehyde	Hexanal	21.36 ± 0.66a	19.87 ± 0.60b	14.27 ± 0.54c	13.26 ± 0.39c
	(E)-2-hexenal	63.60 ± 2.79a	56.18 ± 2.27b	42.38 ± 1.83c	39.71 ± 1.88c
	Heptaldehyde	5.68 ± 0.32a	5.09 ± 0.26a	4.13 ± 0.18b	3.95 ± 0.17b
	Phenylacetaldehyde	1.54 ± 0.10a	1.26 ± 0.08a	–	–
	(E)-2-Heptenal	10.56 ± 0.51b	14.38 ± 1.41a	12.79 ± 0.55a	9.62 ± 0.50b
	Benzaldehyde	2.57 ± 0.13b	3.06 ± 0.16a	3.18 ± 0.18a	3.37 ± 0.17a
	Nonanal	42.39 ± 2.24a	37.60 ± 1.94b	28.92 ± 1.58c	23.27 ± 1.46d
	Decanal	7.68 ± 0.32a	9.28 ± 0.46a	8.74 ± 0.36a	7.95 ± 0.35a
Alcohols	Phenethyl alcohol	9.62 ± 0.42a	7.28 ± 0.32b	6.39 ± 0.24b	4.59 ± 0.27c
	2-Ethylhexanol	5.18 ± 0.27a	5.62 ± 0.29a	4.28 ± 0.23b	3.40 ± 0.22c
	1-Octanol	14.83 ± 0.69a	12.59 ± 0.53b	9.87 ± 0.45c	7.85 ± 0.42d
	Ethanol	13.59 ± 0.73a	12.86 ± 0.60a	7.34 ± 0.38b	3.18 ± 0.20c
	1-hexanol	2.30 ± 0.20a	1.28 ± 0.15b	–	–
Esters	Ethyl acetate	38.60 ± 1.25a	30.82 ± 1.45b	23.71 ± 1.43c	17.92 ± 1.38d
	Ethyl benzoate	3.09 ± 0.18a	3.26 ± 0.20a	2.95 ± 0.12a	2.78 ± 0.14a
	Methyl salicylate	2.83 ± 0.15a	2.47 ± 0.11a	–	–
Ketones	1-Octen-3-one	12.54 ± 0.79a	13.19 ± 0.81a	12.25 ± 0.68a	10.96 ± 0.69a
	Damascenone	9.65 ± 0.39a	7.42 ± 0.34b	5.38 ± 0.28c	4.19 ± 0.21c
	2-Octanone	–	–	2.15 ± 0.12a	2.39 ± 0.14a
Terpenes	D-Limonene	13.52 ± 0.79c	16.79 ± 0.89b	20.56 ± 1.08a	22.85 ± 1.21a
	Linalool	12.80 ± 0.55a	10.57 ± 0.51b	6.79 ± 0.34c	5.86 ± 0.37c
	α-Terpineol	5.19 ± 0.27a	5.32 ± 0.31a	4.96 ± 0.20a	4.67 ± 0.22a
	Geraniol	5.57 ± 0.27a	3.29 ± 0.16b	–	–
	Myrcene	2.59 ± 0.22a	–	–	–
Acids	Acetic acid	5.89 ± 0.34c	7.32 ± 0.36b	8.45 ± 0.41a	9.16 ± 0.43a
	Hexanoic acid	3.29 ± 0.14b	3.86 ± 0.18a	4.09 ± 0.20a	4.58 ± 0.23a
Others	Valeric anhydride	–	–	2.59 ± 0.21a	3.16 ± 0.24a
	2-Pentylfuran	3.58 ± 0.21a	2.63 ± 0.16b	2.45 ± 0.14b	2.19 ± 0.14b

The data are represented by the mean ± standard error. “-” indicates that the substance was not detected Different lowercase letters indicate significant different at 0.05 level. The same below.

The changes in the content of alcohols showed that the contents of phenethyl alcohol, 1-octanol and ethanol in the fruits were significantly reduced by the swelling agent treatment; however, the effect of swelling agent treatment on the content of 2-ethylhexanol was minimal, and the differences between treatments did not reach a significant level (P>0.05).

The changes in the content of esters and ketones showed that the content of ethyl acetate and dammarone in the fruits was significantly reduced under the treatment with swelling agent, while the content of ethyl benzoate and 1-octen-3-one did not change much.

Analysis of the changes in terpene and acid contents showed that the contents of D-limonene, acetic acid and hexanoic acid were significantly increased and the contents of linalool and geraniol were decreased under the swelling agent treatment, but the effect of swelling agent treatment on α-terpineol was minimal. In addition, 2-pentylfuran content in fruits showed a significant decrease under the swelling agent treatment, with the largest decrease in treatment C, which was 38.82% compared to the CK.

### 3.3 E-Nose detection results of fruit

#### 3.3.1 The classification results based on linear discriminant analysis (LDA)

##### 3.3.1.1 Classification results based on maximum values

**Figure 5:**
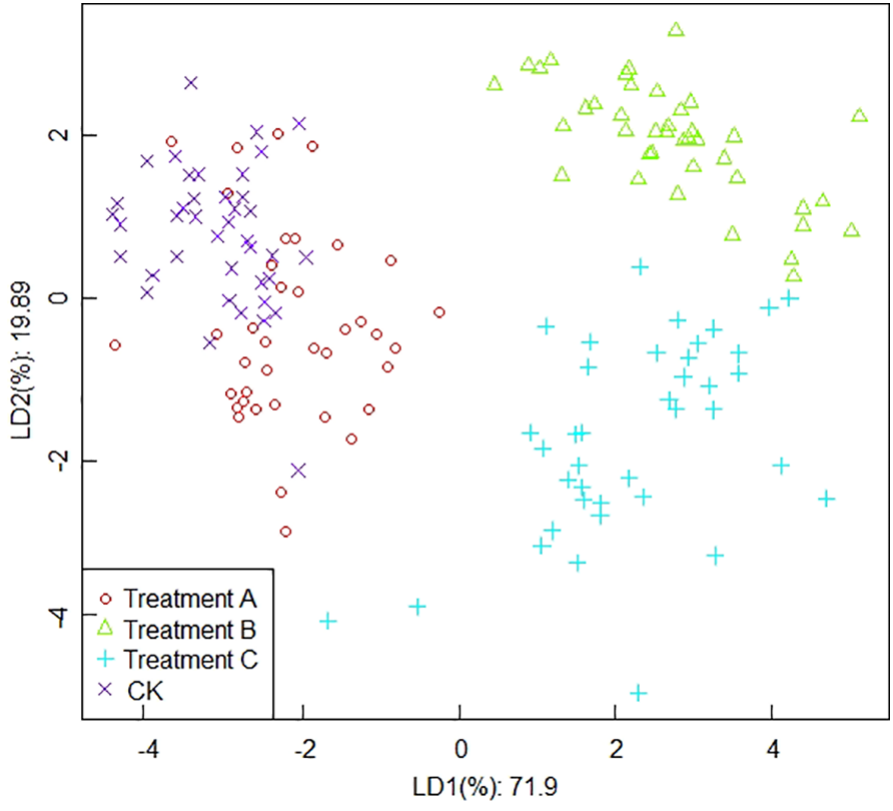
The results of linear discriminant analysis based on the maximum values.

The results of the linear discriminant analysis based on the maximum values were shown in **Figure 5**. It can be seen from **Figure 5** that the first two discriminant functions explain 71.9% and 19.89% of the effect, respectively, with an overall contribution of 91.79%. The LDA emphasizes both the spatial distribution of grape aroma components and the distance between them. The higher the scatter between data collection points, the higher the differentiation of the population. Generally, grape samples from each treatment were separated, but data collection points for treatment A and CK still partially overlapped, which indicated that fruit volatile gases were similar for treatments A and CK, and samples from treatments A and CK may have been incorrectly classified as adjacent groups. There were no obvious overlapping parts between treatment B and treatment C, and they were spaced apart from the others. The distinction between different treatments was more obvious, but the data points collected were more scattered.

##### 3.3.1.2 Classification results based on average values

**Figure 6:**
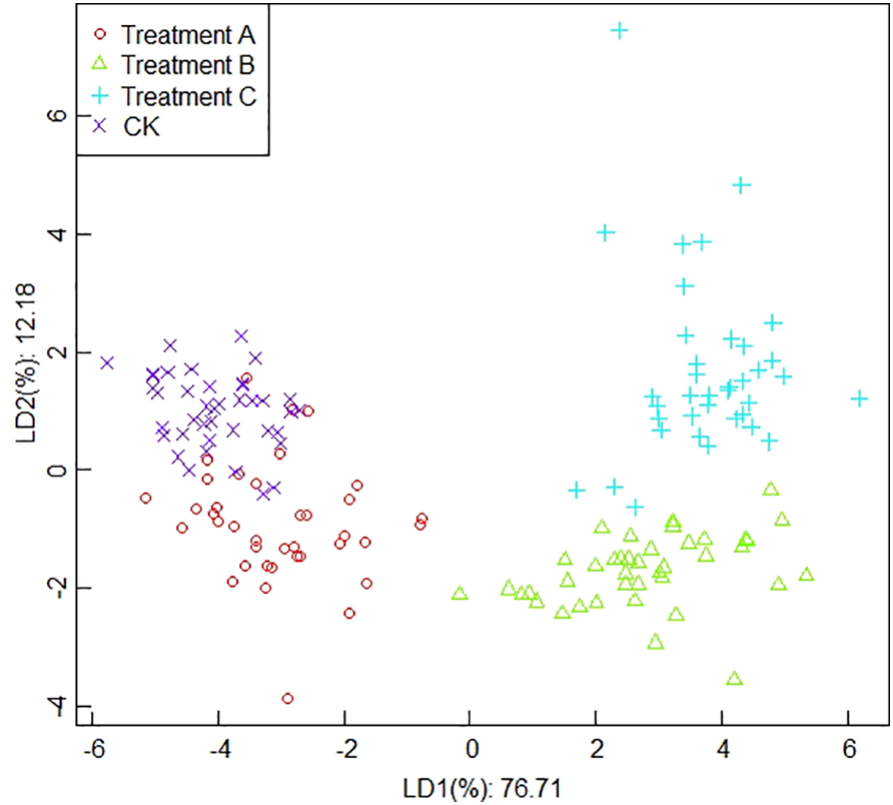
The results of linear discriminant analysis based on the average values.

The analysis results in **Figure 6** indicated that the contribution of the first linear discriminant (LD1) and the second linear discriminant (LD2) of LDA were 76.71% and 12.18%, respectively, and the total contribution of LD1 and LD2 was 88.89%. The two-dimensional scatter plot of the test samples showed that the average data point distribution was relatively concentrated compared to the results of the maximum value-based analysis. The data points of treatment A and the CK have overlapping parts; therefore, it is easy to cause misclassification phenomenon. In contrast, the data points between treatment B and treatment C were relatively close, but there was no obvious overlapping part, and there was a clear distinction between the two, and the interval with other treatments was larger and easy to distinguish.

##### 3.3.1.3 Classification results based on integral values

**Figure 7:**
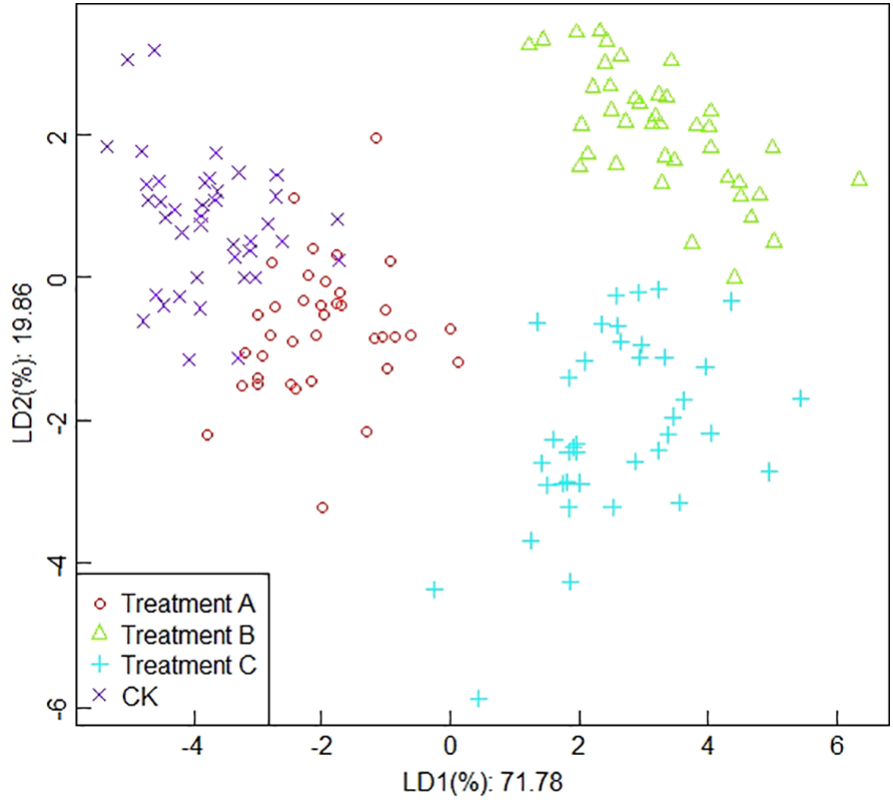
The results of linear discriminant analysis based on the integral values.

It is shown in **Figure 7** that the results of the linear discriminant analysis based on the integrated values. From **Figure 7**, it can be seen that the contribution of the first linear discriminant (LD1) and the second linear discriminant (LD2) of LDA are 71.78% and 19.86%, respectively, and the total contribution of LD1 and LD2 is 91.64%. The CK has a more obvious distinction from treatment B and treatment C, but there is an overlapping part between the CK and treatment A, which does not get a more obvious distinction. In addition, the data collection points of treatment B mainly showed a concentrated trend, while treatment C showed a discrete state, which may be caused by the complexity of fruit volatiles or some measurement errors generated in the experiment, such as the extraction of sample aroma and improper operation during the assay.

##### 3.3.1.4 Classification results based on wavelet transform values

**Figure 8:**
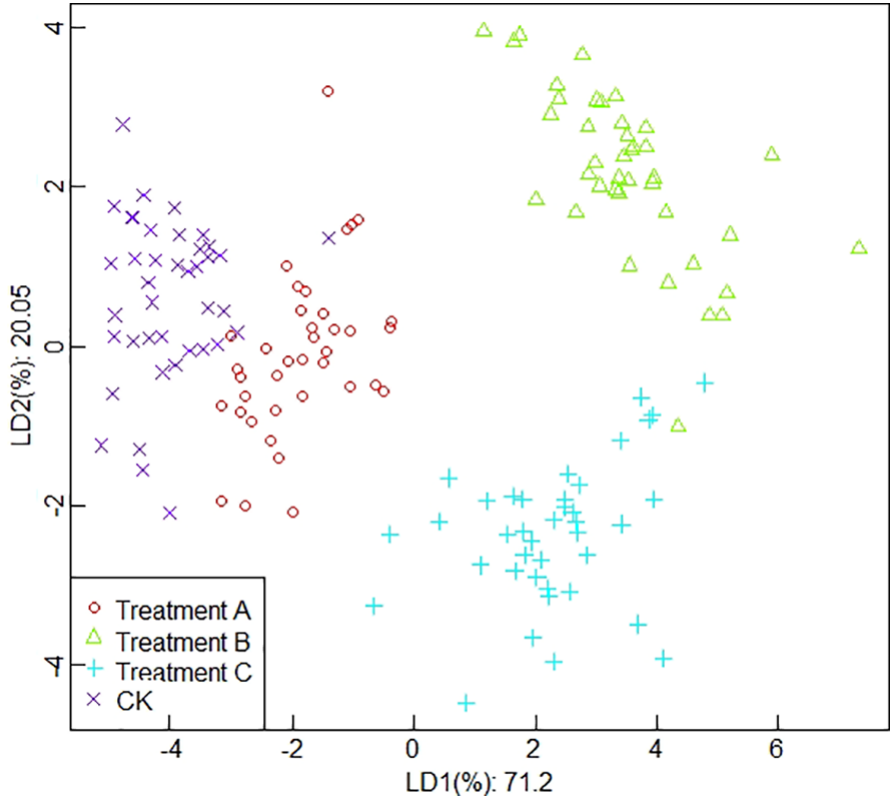
The results of linear discriminant analysis based on wavelet transform values.

Linear discriminant analysis results based on wavelet transform values are shown in **Figure 8**. From **Figure 8**, it can be seen that the contribution of linear discriminant LD1 and LD2 in the analysis of LDA based on wavelet transform values of electronic nose response data is 71.2% and 20.05%, respectively, and the total contribution is 91.25%. From the results of LDA based on the wavelet transform extracted values of the e-nose response data, it can be seen that there is no obvious overlapping part of data points between CK and treatment A, but the data points are close to each other, and there is also no obvious overlapping part between treatment B and treatment C, compared with the first three sets of data. The data points between different treatments were all relatively dispersed, with obvious demarcation lines, and the differentiation was relatively good.

#### 3.3.2 The classification results based on SVM and RF

**Figure 9:**
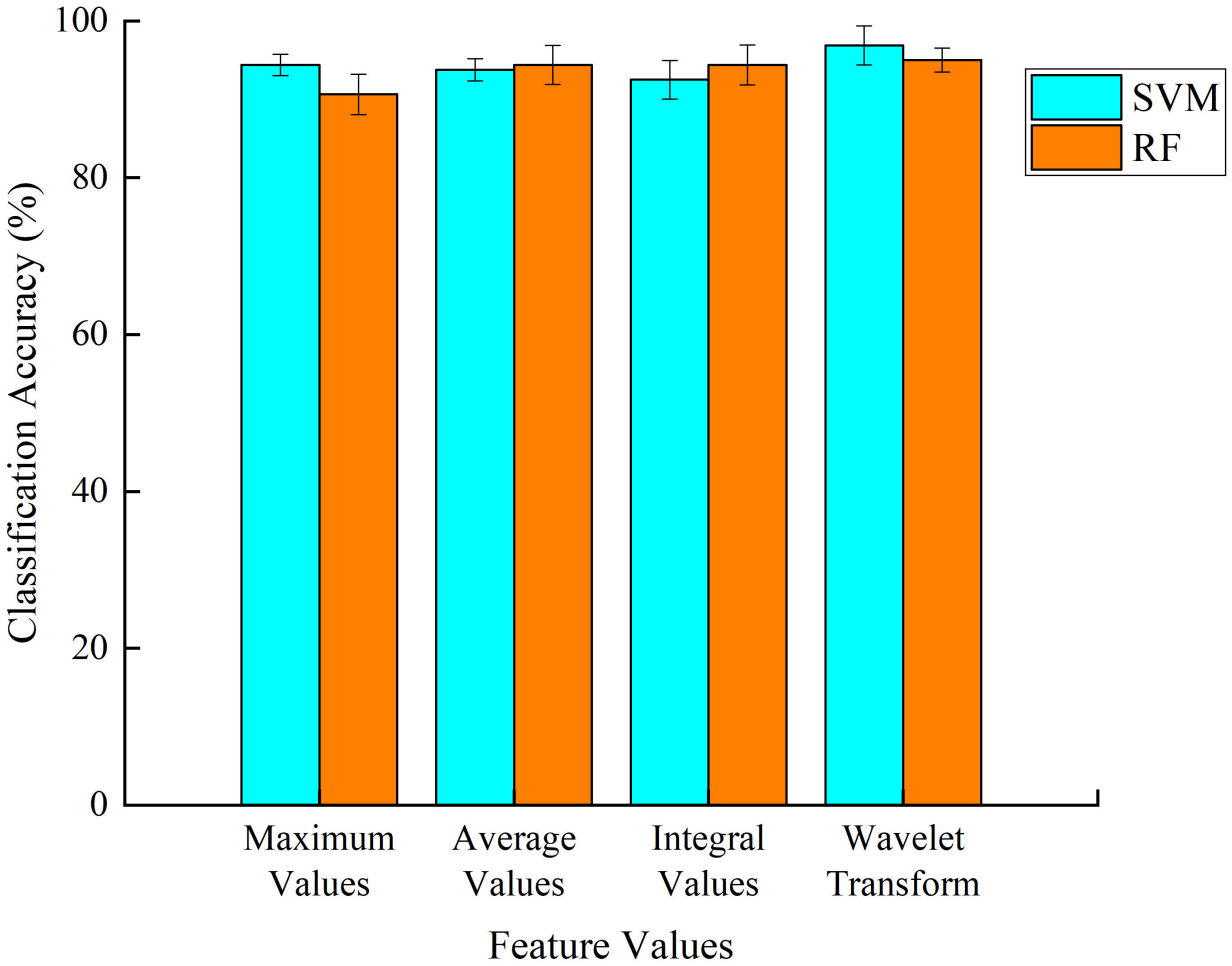
Recognition results of support vector machine (SVM) and random forest (RF) based on different feature extraction methods.

In analyzing the data and constructing the model, the tenfold cross-validation method was used for the sample data. The recognition results of SVM and RF based on different feature extraction methods can be seen in **Figure 9**. The recognition results of SVM based on maximum, mean, integral and wavelet transformed values were 94.38%, 93.75%, 92.5% and 96.88% respectively. While the recognition results of RF based on maximum, mean, integral and wavelet transformed values were 90.63%, 94.38%, 94.38% and 95%, respectively. From **Figure 9**, it is observed that the recognition based on the integral value did not reach the significance level between SVM and RF (P>0.05), while the recognition based on the maximum value, average value and wavelet transform showed a significant difference between SVM and RF ((P>0.05). From the results in **Figure 9**, it can be seen that SVM outperformed RF in performing grape expansion fruit recognition based on various feature extraction methods, and the average recognition accuracy of SVM was 94.4%.

## 4 Discussion

Flavor is one of the key factors in measuring grape quality and depends on a combination of sugars, acids and aromas of the fruit, but Aroma is composed of a variety of volatile compounds, and the formation of fruit volatiles is a dynamic process, with aroma components closely related to fruit quality, processing characteristics and nutritional value. There is a wide variety of aromatic substances in grapes, and their concentration and interactions give grapes their different flavors, mainly aldehydes, alcohols, acids, esters, terpenoids and their derivatives. Factors affecting grape aroma are both endogenous and exogenous, including endogenous factors such as variety, fruit maturity, vine age and rootstock; exogenous factors include environmental conditions, plant growth regulators, etc. These factors interact with each other, resulting in differences in the aroma composition of the grapes and affecting the quality of the berries. The results of this study showed significant changes in the content of aldehydes, alcohols, esters, and ketones in grapes treated with swelling agent, which leads to the assumption that the flavor of fruit treated with swelling agent is slightly inferior to that of naturally ripened fruit, which is consistent with the results of the relevant quality indicators of grapes in this study. Through the determination of soluble sugar, titratable acid, and sugar-acid ratio in the different treated grapes, it was found that the soluble sugar content, titratable acid content, and sugar-acid ratio in naturally ripe fruit differed significantly from those in grapes treated with swelling agent, and soluble sugar, titratable acid, sugar-acid ratio, and volatile substances were the main factors affecting the flavor quality of grapes (Costa et al., 2011), the combined results suggest that changes in nutrients and volatile compounds may affect the overall quality of the grapes, which also proved that the flavor quality of naturally ripened fruits is better than those treated with swelling agent. Fang et al. (Fang et al., 2002) concluded that the use of appropriate amounts of swelling agent enhanced fruit competition for photosynthetic products, accelerated cell division, promoted fruit expansion and altered fruit nutrient composition. Cruz-Castillo et al. (Cruz-Castillo et al., 2014) suggested that swelling agent affect the changes in the content of various sugars and titratable acids during fruit development. The results of this paper show that the nutrient composition of grapes treated with swellings is indeed altered, which is consistent with the results of previous studies. Grapes treated with swelling agent undergo changes in nutrients and volatile compounds, and these changes can have an impact on the sensory experience of the grapes in terms of shape, size, color and flavor. In future research, more in-depth and systematic studies are required in terms of a comprehensive evaluation of how the swelling agent affects the samples and combining sensory evaluation with electronic nose technology. In addition, the quality parameters covered in this study were only the conventional physicochemical parameters such as soluble sugars and titratable acids, therefore, in order to study the quality of grapes (or other varieties of fruits) in more depth, physicochemical parameters such as antioxidant substances and microbial colony counts can be added in future studies. Moreover, the present study was carried out using ‘ Xiangti ‘ grapes as test material, and in future studies, such treatments will be carried out on other grape varieties to study the reactions produced by different grape varieties, as well as to study the reactions that other swelling agent will produce on the grapes.

After the rapid detection of volatile gases in different treated grape fruits using the electronic nose technique, the analytical results showed that grape fruit samples have different accuracy in different pattern recognition processes, and the results of the pattern recognition also demonstrate the potential of the electronic nose technique in distinguishing whether grapes are treated with swelling agent or not. The electronic nose technique can be used to distinguish between wines made from different raw materials (Capone et al., 2013). These results show that the e-nose technique combined with appropriate pattern recognition methods can be a fast and effective means of identifying volatile substances in grape berries. The electronic nose used in this paper is equipped with 16 sensors, and the response values of the sensors are different when detecting volatile substances in different treatments of grape fruits, thus generating response patterns of volatile gases in different treatments of grape samples. However, the electronic nose used in this study has a limited selection of sensors, such as S3 (WSP2110 aldehydes), S4 (MP135 alcohols) and S8 (TGS2620 most volatile organic compounds). Because the results of electronic nose detection are not information about one or more components in the sample, it is the overall information of volatile substances (fingerprint). At the same time, the limited type and number of sensors will affect the accuracy of the model construction. Therefore, in future research, the sensor part of the e-nose can be composed of an array of gas-sensitive sensors with different selectivities, which can be used to analyze mixed gases by taking advantage of their cross-sensitivity to multiple gases and converting the effects of different odour molecules on their surfaces into time-dependent measurable groups of physical signals that can be conveniently computed, as well as designing and preparing e-nose instruments that are specific and selective for volatile compounds in fruits. In addition, in practical applications, different detection methods and environments may also have an impact on the response signal of the electronic nose. and environment will also affect the response signal of the electronic nose, and it is necessary to correct the response signal of the electronic nose and processing, so as to realize the real-time detection of the internal quality of the fruit by the electronic nose.

In summary, LDA had the highest contribution of 91.79% in the cumulative analysis based on maximum values. And combined with the LDA two-dimensional scatter plot analysis of the four eigenvalues, it can be seen that the data collection points of treatment A and CK are close and partially overlapped. Therefore, the electronic nose combined with the LDA method was less effective in practice, probably due to the incomplete quality information contained in the samples and the weaker than expected correlation of the electronic data with the main volatile substances of the fruits. In future work, we will perform more detailed analysis of gas volatiles changes in swelling agent-treated fruits by gas chromatography-mass spectrometry (GC-MS) and e-nose, and then select a smaller number of gas sensors for monitoring swelling agent-treated fruits and develop a dedicated e-nose system for fruit monitoring, which will lay the foundation for further development of identification and classification models of swelling agent-treated grape fruits, and also have important reference significance for quality and safety testing of agricultural products.

## 5 Conclusions

In this study, traditional physical and chemical test detection and electronic nose techniques were used to detect and classify and identify unexpanded and swollen-treated fruits, with the following main conclusions:

1. Through analyzing the differences in quality indicators such as soluble sugar and titratable acid between naturally ripened fruits and those treated with swelling agent, the results show that: naturally ripened grapes have better flavor and it is possible to distinguish whether the grapes are treated with swelling agent or not based on the results, but the operation process is tedious, time-consuming, requires the participation of professional testers and cannot be realized in real time. In contrast, the use of an electronic nose system based on a metal oxide semiconductor sensor array is faster and more convenient for practical applications.

2. By comparing the performance of LDA, SVM and RF modeling, the feature matrices suitable for distinguishing whether grapes were treated with swelling agent were preferentially selected, and the classification models and for different treatments of grapes were constructed. The classification results showed that the two-dimensional scatter plot results obtained by LDA based on different feature values were 91.79%, 88.89%, 91.64% and 91.25%, but there was overlap in the classification process, which led to the samples not being accurately classified and prone to errors in practical applications. In contrast, the classification accuracy of both nonlinear recognition methods SVM and RF is higher than that of LDA, and when pattern recognition is performed based on different feature values, SVM has a better recognition effect with an average recognition accuracy of 94.4%. It indicates that nonlinear recognition methods are more suitable than linear recognition methods to solve nonlinear recognition classification problems, such as the recognition of whether grapes were treated with swelling agent or not.

Based on the results of the above study, the analysis of the e-nose response data can be used to discriminate between naturally ripe and CPPU treated grapes. The model was successfully developed for the identification of naturally ripe and CPPU-treated grapes. Evidence is provided in this study that electronic noses can be used as a non-destructive method to identify swelling agent-treated fruits.

## Data availability statement

The raw data supporting the conclusions of this article will be made available by the authors, without undue reservation.

## Author contributions

JQ: Conceptualization, Writing – original draft. GS: Investigation, Methodology, Visualization, Writing – original draft. LY: Methodology. LW: Conceptualization, Data curation, Funding acquisition, Project administration, Validation, Writing – review & editing. XW: Resources, Writing – review & editing. SL: Formal analysis, Software, Validation. YF: Supervision, Writing – review & editing. DJ: Formal analysis. YC: Investigation. YM: Formal analysis, Software.
